# LincRNA*FEZF1-AS1* represses *p21 expression to promote* gastric cancer proliferation *through LSD1-Mediated H3K4me2 demethylation*

**DOI:** 10.1186/s12943-017-0588-9

**Published:** 2017-02-16

**Authors:** Yan-wen Liu, Rui Xia, Kai Lu, Min Xie, Fen Yang, Ming Sun, Wei De, Cailian Wang, Guozhong Ji

**Affiliations:** 1 Department of Oncology, Zhongda Hospital, Medical School, Southeast University, Nanjing, Jiangsu People’s Republic of China; 2 Department of Laboratory, Affiliated Chest Hospital of southeast University, Nanjing, Jiangsu People’s Republic of China; 3 Department of surgery, Affiliated the second hospital of Bengbu Medical College, Lianyungang, jiangsu People’s Republic of China; 4 Department of Biochemistry and Molecular Biology, Nanjing Medical University, Nanjing, Jiangsu People’s Republic of China; 5 Department of Gastroenterology Second Affiliated Hospital of Nanjing Medical University, Nanjing, Jiangsu People’s Republic of China

**Keywords:** *FEZF1-AS1*, LSD1, H3K4me2, P21, Gastric cancer

## Abstract

**Background:**

Although the prognosis of gastric cancer patients have a favorable progression, there are some patients with unusual patterns of locoregional and systemic recurrence. Therefore, a better understanding of early molecular events of the disease is needed. Current evidences demonstrate that long noncoding RNAs (lncRNAs) may be an important class of functional regulators involved in human gastric cancers development. Our previous studies suggest that *HOTAIR* contributes to gastric cancer development, and the overexpression of *HOTAIR* predicts a poor prognosis. In this study, we investigated the characteristic of the LncRNA FEZF1-AS1 in gastric cancer.

**Methods:**

QRT-PCR was used to detect the expression of FEZF1-AS1 in gastric cancer tissues and cells. MTT assays, clonogenic survival assays and nude mouse xenograft model were used to examine the tumorigenesis function of FEZF1-AS1 in vitro and in vivo. Bioinformatics analysis were used to select downstream target genes of FEZF1-AS1. Cell cycle analysis, ChIP, RIP,RNA Pulldown assays were examined to dissect molecular mechanisms.

**Results:**

In this study, we reported that *FEZF1-AS1*, a 2564 bp RNA, was overexpressed in gastric cancer, and upregulated *FEZF1-AS1* expression indicated larger tumor size and higher clinical stage; additional higher expression of *FEZF1-AS1* predicted poor prognosis. Further experiments revealed that knockdown *FEZF1-AS1* significantly inhibited gastric cancer cells proliferation by inducing G1 arrest and apoptosis, whereas endogenous expression *FEZF1-AS1* promoted cell growth. Additionally, RIP assay and RNA-pulldown assay evidenced that *FEZF1-AS1* could epigenetically repress the expression of P21 via binding with LSD1, the first discovered demethylase. ChIP assays demonstrated that LSD1 could directly bind to the promoter of P21, inducing H3K4me2 demethylation.

**Conclusion:**

In summary, these data demonstrated that *FEZF1-AS1* could act as an “oncogene” for gastric cancer partly through suppressing P21 expression; *FEZF1-AS1* may be served as a candidate prognostic biomarker and target for new therapies of gastric cancer patients.

**Electronic supplementary material:**

The online version of this article (doi:10.1186/s12943-017-0588-9) contains supplementary material, which is available to authorized users.

## Background

Gastric cancer is the third leading cause of cancer-related deaths worldwide, and the poor prognosis of patients is largely due to the high frequency of tumor recurrence or metastasis within 24 months after surgical resection [1, 2]. To improve gastric cancer early diagnosis and targeted therapy, a better understanding of early molecular events of the disease is warranted. Cell proliferation is a pivotal characteristic of malignancy and a hallmark cancer capability [3]. Dysregulation of all cycle is a vital reason for tumor cell proliferation. Moreover, the cell cycle regulation has come to be a promising therapeutic target, which suggests that discovery of novel proliferation related genes could lead to improve treatment of cancer [4, 5].

Recent integrative genomic studies have revealed that 98% of the human genome transcripts are non-coding RNA (ncRNA) with limited or no protein-coding capacity [6–8]. Long non-coding RNAs (lncRNAs), greater than 200 nt are important new members of the ncRNA family [9]. Researchers have demonstrated that the aberrant lncRNAs expression involve in diverse human diseases, in particular cancers [10–12]. Such one is *HOTAIR,* lots of studies have shown that *HOTAIR* is overexpressed in colorectal cancer, pancreatic cancer, breast cancer, gastric cancer and gastrointestinal stromal tumors and is positively correlated with a poor clinical outcome [13–16]. Furthermore, lncRNA regulate drug resistance, for instance, H19 epigenetically inducted MDR1-associated drug resistance in human hepatocellular carcinoma cells [17]. Recently, a study showed that nearly 76% of the GENCODE annotated lncRNAs was differentially expressed between gastric cancer and normal gastric tissue [18]; for example, HOTAIR and HOXA-AS2 were overexpressed in gastric cancer and indicated poor prognosis; however, a large number of lncRNAs have been uncharacterized [19–22].

Recently, mounting evidences showed that some lncRNAs epigenetically regulate gene expression by DNA methylation and histone modifications, which contain methylation, acetylation, phosphorylation et al. [23]. Histone methylation is Histone H3/H4 on lysine different sites methylation or demethylation, which is regulated by histone methylases or demethylases. *HOTAIR* and *ANRIL* etc. could recruit and bind with the Polycomb complex PRC2 (EZH2, SUZ12 and EED), which enhances histone H3lysine-27 trimethylation, affecting chromatin compression tightness in suppressing gene expression [15, 24]. Lysine-specific demethylase 1(LSD1) is the first discovered demethylase, which demethylates mono— and di-methylated residues of lysine-4 on histone H3 (H3K4me1, H3K4me2 orH3K9me1) and results in transcriptional repression [25, 26]. In addition, LSD1 also activates transcription through demethylation of H3K9me2 [27]. LSD1 is pivotal for mammalian tumorigenicity and progression in many type of cancers, moreover, LSD1 overexpression predict poor prognosis and aggressive tumor biology [28–31]. Many studies had shown LSD1 epigenetically regulate cell cycle related gene expression to affect G1/S phase arrest, contributing to cell proliferation [32–34].

*FEZF1-AS1* is an lncRNA producing a 2564 bp transcript, located in chromosome 7. In this study, we demonstrated that *FEZF1-AS1* was overexpressed in the tumor tissues than the paracancerous tissues; furthermore, overexpression of *FEZF1-AS1* was observed in larger tumors, advanced gastric cancer and predicted poor DFS. Additional experiments revealed that *FEZF1-AS1* knockdown significantly repressed proliferation both in vitro and vivo, and inhibited cells cycle progression by causing G1/S arrest. In addition, *FEZF1-AS1* also recruited and bound to LSD1 to epigenetically repress downstream gene p21, thereby promoting proliferation in advanced stages of gastric cancer. By these efforts, we aim to propose a model for *FEZF1-AS1*-mediated cell proliferation in gastric cancer.

## Methods

### Tissue samples

In this study, matched tumor tissues and adjacent non-tumor tissues were obtained from 82 gastric cancer patients at the Department of Surgical Oncology Jiangsu Province People’s Hospital, Nanjing Medical University from March 2011 to December 2011. Two pathologists evaluated all specimens according to the World Health Organization (WHO) guidelines and the pTNM Union for International Cancer Control (UICC) pathological staging criteria. No local or systemic treatments were administered to these patients before surgery. The tissues were immediately frozen in liquid nitrogen and stored at −80 °C until use. Informed consent was obtained from all patients. The Human Research Ethics Committee of Jiangsu Province People’s Hospital approved the study.

### Total RNA extraction Quantitative real-time polymerase chain reaction

Total RNA was extracted from the cultured cells and frozen tissues using TRIzol reagent (Invitrogen, Karlsruhe, Germany) following the manufacturer’s protocol. Quantitative real-time polymerase chain reaction (PCR) was performed to detect *FEZF1-AS1* and *P21* using the PrimeScript RT Reagent Kit and SYBR Premix Ex Taq (TaKaRa, Dalian, China) according to the manufacturer’s instructions. The results were normalized to the expression of glyceraldehyde-3-phosphate dehydrogenase (GAPDH). The specific primers used are presented in Additional file 1: Table S1. The qPCR results were analyzed and expressed relative to the CT (threshold cycle) values and then converted to fold changes.2.0-fold change was considered significant.

### Plasmid generation

The *FEZF1-AS1* sequence was synthesized and subcloned into the pCDNA3.1 (Invitrogen, Shanghai, China) vector. Ectopic expression of *FEZF1-AS1* was achieved via pCDNA-FEZF1-AS1 transfection, with an empty pCDNA3.1 vector used as a control. We also synthesised shRNA sequence targeted *FEZF1-AS1*. Si-FEZF1-AS1 sequence removed five bases of the 3 ‘end were converted to sh-FEZF1-AS1. After annealing of the complementary shRNA oligonucleotides, we cloned the annealed oligonucleotides into pENTR vector (sh-*FEZF1-AS1*) (Additional file 1: Table S1). The expression levels of *FEZF1-AS1* were detected by qPCR.

### Cell culture

The MGC-803 lines were cultured in RPMI 1640 medium containing 10% fetal bovine serum and incubated at 37 °C, 5% CO_2_, and saturated humidity. The SGC-7901 cells were cultured in DMEM medium containing 10% fetal bovine serum and incubated at 37 °C, 5% CO2, and saturated humidity. The AGS lines were cultured in F 12 medium containing 10% fetal bovine serum and incubated at 37 °C, 5% CO_2_, and saturated humidity. Cell growth was observed under an inverted microscope. Cells in the logarithmic growth phase were harvested for the experiments.

### Cell transfection

Plasmid vectors (pCDNA3.1-FEZF1-AS1 and pCDNA3.1) for transfection were prepared using DNA Midiprep or Midiprep kits (Qiagen, Hilden, Germany) and transfected into MGC-803cells. The si-FEZF1-AS1, sh-FEZF1-AS1, si-LSD1 or si-NC was transfected into AGS and SGC-7901 cells (Additional file 1: Table S1).

### Cell cycle and apoptosis analysis

AGS and SGC-7901cells transiently transfected with si-FEZF1-AS1 or si-NC and MGC-803 transfected with pcDNA-FEZF1-AS1 or pcDNA-3.1, cells were analyzed by flow cytometry (FACScan; BD Biosciences) using CellQuest software (BD Biosciences).

### MTT assay and clone formation

MTT assay and clone formation were used for evaluated cell viability and proliferation. Cell proliferation was documented following the manufacturer’s protocol every 24 h. For the colony formation assay, cells were seeded in a fresh six-well plate and maintained in media containing 10% FBS, replacing the medium every 4 days. After 14 days, methanol and stained with 0.1% crystal violet (Sigma-Aldrich) fixed cells and count clones.

### Tumor formation assay in a nude mouse model

The male athymic BALB/c nude mice aged 5 weeks were maintained under specific pathogen-free conditions and manipulated according to protocols approved by the Shanghai Medical Experimental Animal Care Commission. A volume of 0.1 ml of suspended cells with sh-FEZF1-AS1 and pENTR vector (EV) was respectively subcutaneously injected into the posterior flank of each mouse. At 15 days post-injection, mice were euthanized and the primary tumors were excised, paraffin-embedded, formal infixed and performed H&E staining, immunostaining analysis for Ki-67 protein expression.

### Western blotting analysis and antibodies

Cell lysates were prepared using RIPA protein extraction reagent (Beyotime, Beijing, China) supplemented with a protease inhibitor cocktail (Roche, CA, USA) and phenylmethylsulfonyl fluoride (Roche). GAPDH was used as a control. Antibodies (1:1000) against cyclin D1, CDK2, CDK4, CDK6 and P21were purchased from Abcam.

### Subcellular fractionation location

The separation of nuclear and cytosolic fractions was performed using the PARIS Kit (Life Technologies) according to the manufacturer’s instructions.

### Chromatin immunoprecipitation (ChIP)

We performed chromatin immunoprecipitation (ChIP) using the EZ ChIP™Chromatin Immunoprecipitation Kit for cell line samples (Millipore, Bedford, MA). Briefly, we sonicated the crosslinked chromatin DNA into 200- to 500-bp fragments. The chromatin was then immunoprecipitated using an anti-demethyl-histone H3 antibody and LSD1 (1:1000). Normal mouse IgG was used as the negative control. The primer sequences are listed in Additional file 1: Table S1. The antibodies for the ChIP assays of LSD1, H3K4 and H3K9 were obtained from Millipore. Quantification of the immunoprecipitated DNA was performed using qPCR with SYBR Green Mix (Takara). The ChIP data were calculated as a percentage relative to the input DNA using the equation 2[Input Ct- Target Ct] × 0.1 × 100.

### RNA immunoprecipitation(RIP)

We performed RNA immunoprecipitation (RIP) experiments using the Magna RIP™RNA-Binding Protein Immunoprecipitation Kit (Millipore, USA) according to the manufacturer’s instructions. The antibodies for the RIP assays of LSD1 were obtained from Abcam. The co-precipitated RNAs were detected by reverse-transcription PCR. The total RNAs were the input controls.

### RNA pulldown assay

Biotin-labeled RNAs were transcribed in vitro with the Biotin RNA Labeling Mix (Roche Diagnostics) and T7 RNA polymerase (Roche Diagnostics), treated with RNase-free DNase I (Roche), and purified with an RNeasy Mini Kit (Qiagen, Valencia, CA). Next, 1 mg whole-cell lysates from SGC7901 cells was incubated with 3 μg of purified biotinylated transcripts for 1 h at 25 °C. Complexes were isolated with streptavidin agarose beads (Invitrogen). The beads were washed briefly three times and boiled in sodium dodecyl sulfate (SDS) buffer, and the retrieved protein was detected using the standard western blot technique.

### Bioinformatics methods

Gene set enrichment analysis (GSEA) software was downloaded from Broad Institute (http://www.broadinstitute.org/gsea/index.jsp). Gene profiling data downstream *FEZF1-AS1* were obtained from Gene Expression Omnibus (GEO) site (http://www.ncbi.nlm.nih.gov/geo/query/acc.cgi?acc=GSE53137). Significantly enriched gene sets were identified, which produced a nominal *P*-value 0.05. UCSC Genome Browser (http://genome.ucsc.edu/cgi-bin/hgGateway) was used to analyze promoter regions.

### Statistical analysis

The SPSS 17.0 statistical analysis software was used for the statistical analysis of the experimental data. The significance of differences between groups was estimated by Student’s t-test. The levels of *FEZF1-AS1* in the gastric cancer patients were compared using the Mann–Whitney U test. The disease-free survival probability was analyzed using Kaplan-Meier methods and evaluated using the log-rank test. A *p* value less than 0.05 were considered significant.

## Results

### FEZF1-AS1 expression levels in human gastric cancer tissue

**Fig. 1:**
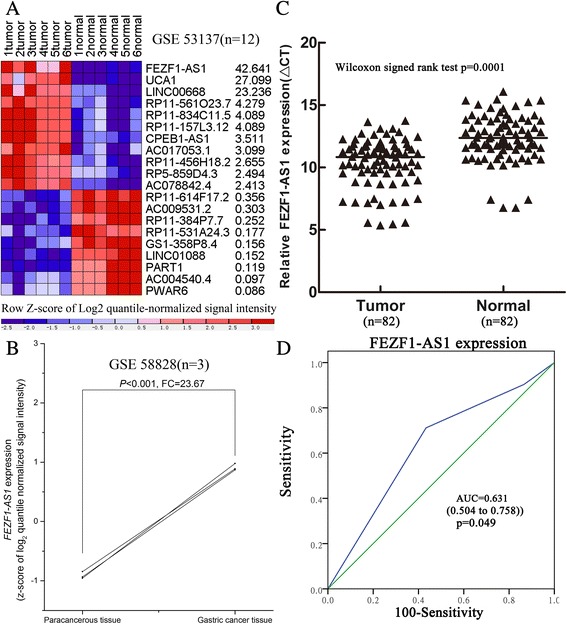
*FEZF1-AS1 expression levels in human gastric cancer tissue.* **a**, **b** Relative expression levels of *FEZF1-AS1* in GEO database. **c** Relative expression of *FEZF1-AS1* in the tumor tissue compared to adjacent normal tissues (*n =* 82). FEZF1-AS1 expression was evaluated by qRT-PCR and normalized to GAPDH expression. **d** ROC curve for prediction of gastric cancer based on *FEZF1-AS1* expression, using corresponding adjacent normal tissues as a control. *P < 0.05

To explore the function of LncRNAs in gastric cancer, firstly, we profiled the expression levels of LncRNAs in human gastric cancer tissues and normal tissue by using raw microarray data downloaded from GEO (GSE53137) [35] and mapsoft (http://lncrnamap.mbc.nctu.edu.tw/php/search.php). The results show that *FEZF1*-AS1 expression level is upregulated in gastric cancerous tissues compared with noncancerous tissues (42.64 Fold, Fig. 1a); furthermore, *FEZF1*-AS1 expression is also overexpressed in gastric cancer tissues (GSE58828)(23.67 Fold, Fig. 1b). Next, we used qRT-PCR to detect *FEZF1-AS1* expression in 82 paired gastric cancer samples and adjacent histologically normal tissues. *FEZF1-AS1* expression was significantly overexpressed in the gastric cancer (*p =* 0.0001) compared to the adjacent histologically normal tissues (Fig. 1c). Furthermore, receiver operating characteristic (ROC) curves were determined to evaluate the sensitivity and specificity of *FEZF1-AS1* expression in predicting gastric cancer tissues from normal tissues. Notably, *FEZF1-AS1*dayisplayed predictive, with an area under curve (AUC) of 0.631 (*P =* 0.049, Fig. 1d). These results implied that *FEZF1-AS1*might act as “oncogene” to promote the progression of gastric cancer and might provide imperative clinical significance in gastric cancer diagnosis.

### FEZF1-AS1 upregulation associated with tumor size, stage and poor survival of gastric cancer patients

**Fig. 2:**
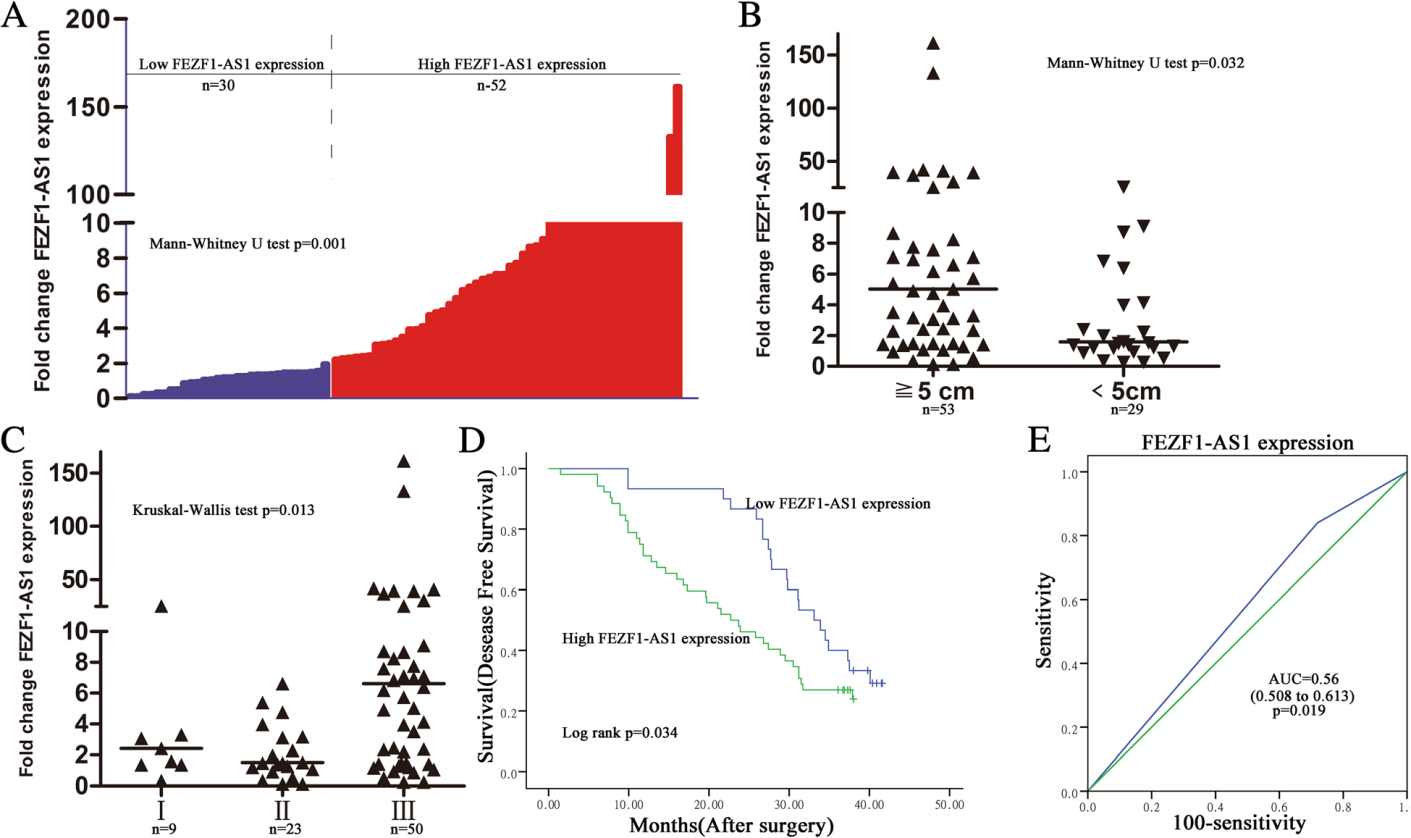
*FEZF1-AS1 upregulation associated with tumor size, stage and poor survival of gastric cancer patients.* **a** *FEZF1-AS1* expression was classified into two groups. Final results were presented as fold change in tumor tissues relative to normal tissues. Fold change is greater than or equal to 2.0 for high expression, and less than 2.0 for low expression. **b**, **c** *FEZF1-AS1* expression was correlation with tumor size and stage. **d** Kaplan–Meier disease-free survival curves according to *FEZF1-AS1* expression levels. **e** ROC analysis of based on *FEZF1-AS1* expression for survival prediction of patients with gastric cancer. *P < 0.05, **P < 0.01

To assess whether *FEZF1-AS1*expression was correlated with clinical pathological parameters and prognosis of gastric cancer, according to relative *FEZF1-AS1* expression in tumor tissues, the 82 gastric patients were classified into two groups: the high *FEZF1-AS1* group (*n =* 52, fold-change ≥2); and the low *FEZF1-AS1* group (*n =* 30, fold-change < 2) (Fig. 2a). The clinical pathology parameters of 82 gastric carcinoma patients were shown in Table 1. Noticeably, high *FEZF1-AS1* expression in gastric cancer was significantly correlated with tumor size and advanced TNM stage (Fig. 2b and c). For disease-free survival patients with high *FEZF1-AS1* expression had a significantly poorer prognosis than those with low *FEZF1-AS1* expression in gastric cancer patients (*P* <0.05, log-rank test; Fig. 2d). Furthermore, ROC curves were determined to evaluate the sensitivity and specificity of the survival prediction based on the *FEZF1-AS1* expression. *FEZF1-AS1* displayed predictive, with an area under curve (AUC) of 0.56 (*P =* 0.019) (Fig. 2e). The AJCC TNM staging system has been widely accepted as a powerful predictor of treatment response and survival in gastric cancer, thus it is of interest to test whether the prognostic value of the FEZF1*-AS1* is independent of AJCC stage. Multivariable Cox regression analysis adjusting AJCC stage and other factors confirmed the association between *FEZF1-AS1* expression and shorter survival (hazard ratio (HR), 0.38; 95% confidence interval (CI), 0.195–0.739; P <0.01). Collectively, these results indicate that *FEZF1-AS1* overexpression play an important role in gastric cancer progression and may be useful for the prognostic or progression markers in gastric cancer.

**Table1:** Correlation of the expression of FEZF1-AS1 with clinicopathologic features in gastric cancer

Characteristics	N (%)	*FEZF1-AS1* ^a^		*p*-value
		High	Low	
Gender				0.528
Male	51(62.20%)	31	20	
Female	31(37.80%)	21	10	
Age				0.670
≤65	27(32.93%)	18	9	
>65	55(67.07%)	34	21	
Stage				0.023*
I	9(10.97%)	5	4	
II	23(28.05%)	10	13	
III	50(60.98%)	37	13	
Tumor size				0.010*
≤5	29(35.37%)	13	16	
>5	53(64.63%)	39	14	
Defferation				0.482
Well	37(45.12%)	25	12	
Poorly	45(54.88%)	27	18	
Lauren type				
Intestinal	35(42.68%)	20	15	0.312
Diffuse	47(57.32%)	32	15	

^a^Fold change(FC) (tumor tissues relative to normal tissues) Fold change is greater than or equal to 2.0 for high expression, and less than 2.0 for low expression

*P < 0.05 was considered significant (Mann–Whitney U test between 2 groups,Kruskal-Wallis H(K) test among 3 groups)

**Table 2:** Transcription factor of promoter of FEZF1-AS1

ModelID	Score	Relativescore	Start	End	predictedsitesequence
MA0079.3	11.569	0.926691945898605	751	761	GCTCCTCCCTT
MA0079.3	11.472	0.925471574849174	1809	1819	TTCCCTCCCTC
MA0079.3	11.472	0.925471574849174	1881	1891	TTCCCTCCCTC
MA0079.3	11.472	0.925471574849174	1913	1923	TTCCCTCCCTC
MA0079.3	11.445	0.925131883938508	1905	1915	CTCCCTCCCTC
MA0079.3	11.445	0.925131883938508	1909	1919	CTCCCTCCCTC
MA0079.3	9.880	0.905442392264696	1798	1808	GCTCCTCCTTT
MA0079.3	9.783	0.904222021215265	1893	1903	TTCCCTCCTTC
MA0079.3	9.756	0.903882330304599	1877	1887	CTCCCTCCTTC
MA0079.3	9.756	0.903882330304599	1901	1911	CTCCCTCCTTC

### Modulation of FEZF1-AS1expression in gastric cancer cells

To investigate the effect of *FEZF1-AS1*on the gastric cancer cells, we firstly examined the endogenous expression levels of *FEZF1-AS1* in various cancer cell lines by qRT-PCR. As shown in Additional file 2: Figure S1A, of the five gastric cancer cell lines (SGC- 7901, BGC-823, MGC-803, AGS and HGC-27), SGC-7901 and AGS expressed higher levels of *FEZF1-AS1* than the normal gastric epithelium cell line (GES-1); however, BGC-823 and MGC-803 expressed deficiency. Therefore, we chose SGC-7901 and AGS as loss of function experimental cell lines and MGC-803 as gain of function experimental cell line. The results showed that *FEZF1-AS1* expression was effectively knocked down in AGS、SGC-7901and MGC-803cells by si-*FEZF1-AS1*1# + 2#、si-*FEZF1-AS1*2# + 3# and pcDNA-FEZF1-AS1 (Additional file 2: Figure S1B), which were subsequently used in the further experiments. The efficiency of the sh- *FEZF1-AS1*、si-LSD1、si-SP1 and pcDNA-SP1 was shown in Additional file 2: Figure S1C, D and E.

### FEZF1-AS1 promoted gastric cancer cells proliferation in vitro and vivo

**Fig. 3:**
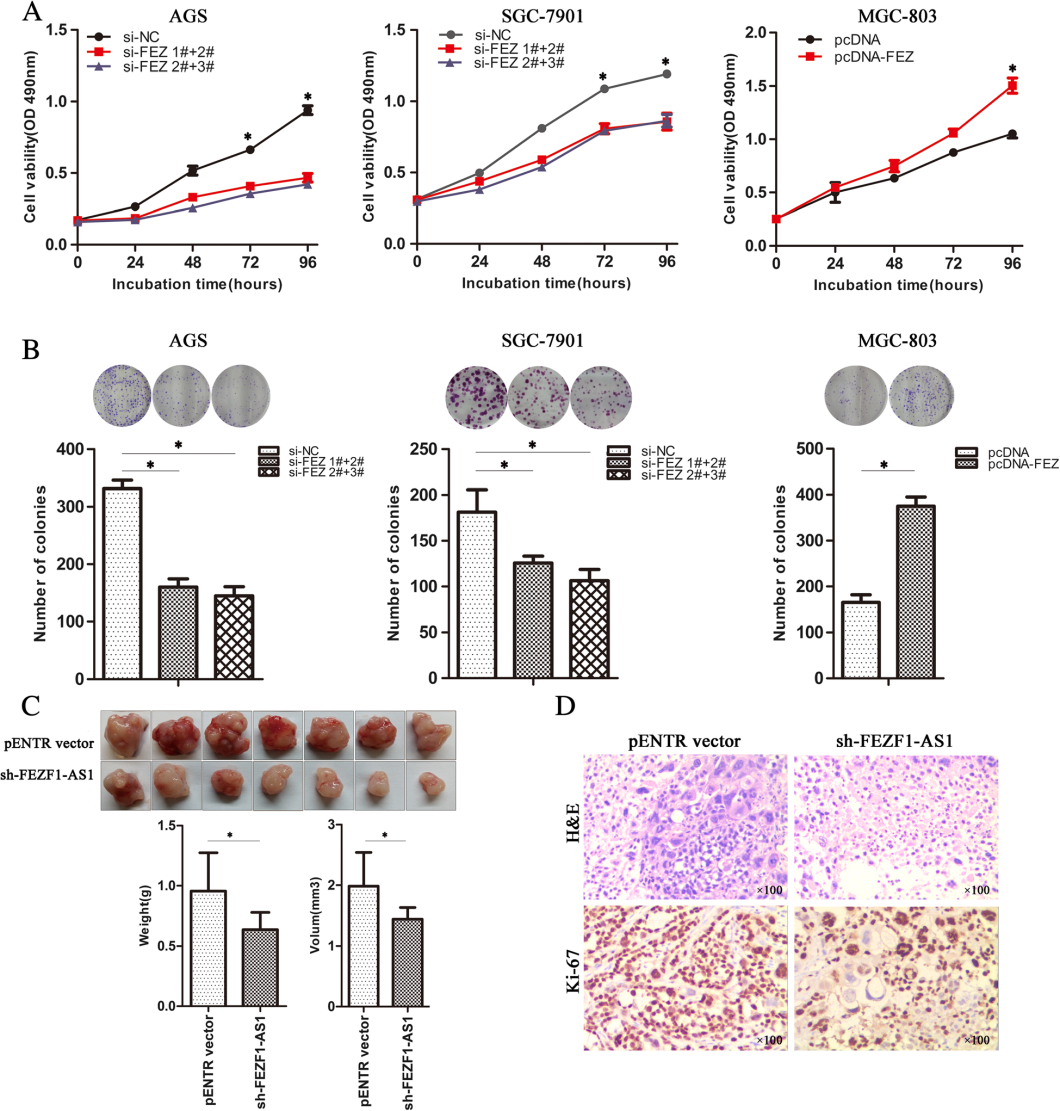
*FEZF1-AS1 promoted gastric cancer cells proliferation* in vitro and vivo*.* **a** MTT assays were used to determine the cell viability for si-FEZF1-AS1 and pCDNA-FEZF1-AS1 transfected AGS, SGC-7901 and MGC-803 cells. Values represented the mean ± SD; from three independent experiments. **b** Colony-forming assays were conducted to determine the proliferation of si-FEZF1-AS1 and pCDNA-FEZF1-AS1 transfected AGS, SGC-7901 and MGC-803 cells. Values represented the mean ± SD; from three independent experiments. **c** Effects of *FEZF1-AS1* downexpression on tumor growth in a xenograft mouse model. Empty vector or sh- FEZF1-AS1 was transfected into SGC-7901 cells, which were injected in the nude mice (*n =* 7), and the tumors were obtained at day 16 and weighed. *P < 0.05 and **P < 0.01. **d** The tumor sections were under H&E staining and IHC staining using antibodies against ki-67

To investigate the effect of *FEZF1-AS1*on the gastric cancer cells, MTT assays were performed and the results revealed that knockdown of *FEZF1-AS1* decreased AGS and SGC-7901 cells proliferation compared with the respective controls, whereas ectopic overexpression *FEZF1-AS1* promoted cell growth in MGC-803 (Fig. 3a). Similarly, the results of colony-formation assays revealed that clonogenic survival was significantly decreased following downregulation of *FEZF1-AS1* in SGC-7901 and AGS cells, but markedly increased in *FEZF1-AS1* overexpression MGC-803 (Fig. 3b). To further investigate the tumorigenesis function of *FEZF1-AS1* in vivo, we used nude mouse xenograft model. Sh- *FEZF1-AS1* stably transfected SGC-7901 cells were injected subcutaneous of fourteen nude mice respectively. Two weeks later, the mice were sacrificed and the xenografts were collected. As expected, the sh- *FEZF1-AS1* group exhibited generally smaller tumors and displayed less weight and volum compared to the pENTR vector group (Fig. 3c). This difference was further confirmed following examination of the xenograft by hematoxylin and eosin (HE), and the tumors developed from sh-*FEZF1-AS1* cells displayed lower Ki-67 staining than control group (Fig. 3d). Taken together, these results indicated that *FEZF1-AS1* possessed a vital role for *FEZF1-AS1*in tumorigenicity and tumor growth of gastric cancer.

### FEZF1-AS1 promoted proliferation of gastric cancer cells by inducing cell-cycle progress and reducing apoptosis in gastric cancer cells

**Fig. 4:**
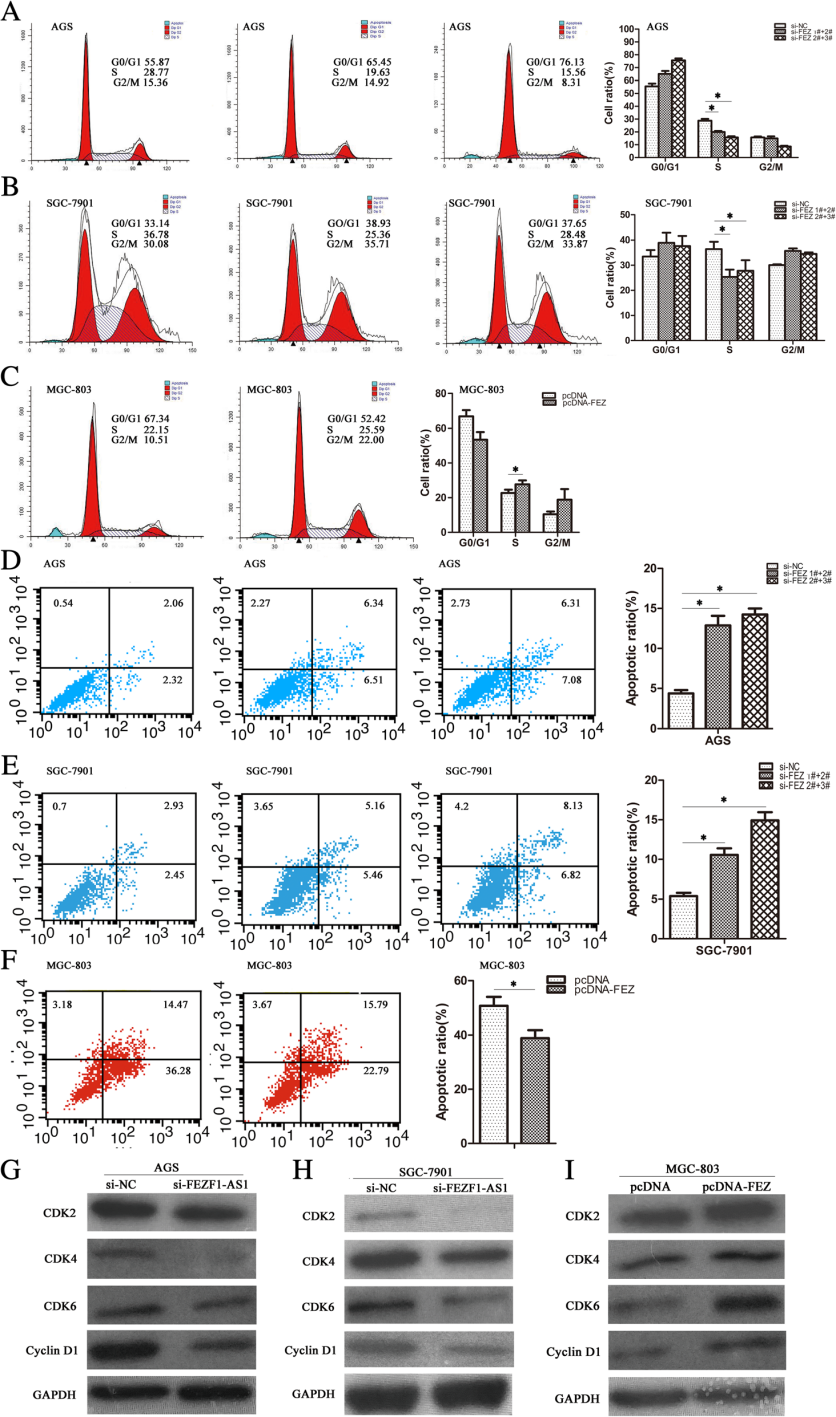
*FEZF1-AS1 promoted proliferation of gastric cancer cells by inducing G1-S and reducing apoptosis in gastric cancer cells.* **a**, **b** and **c** The bar chart represented the percentage of AGS, SGC-7901 and MGC-803 cells in G0/G1, S or G2/M phase, as indicated. **d**, **e** and **f** Flow cytometry was used to detect the apoptotic rates of cells. LR, early apoptotic cells; UR, terminal apoptotic cells. Values represented the mean ± SD, from three independent experiments *P < 0.05, **P < 0.01(**g**, **h** and **i**) Western blot analysis of CDK2, CDK4, CDK6 and CyclinD1 in AGS, SGC-7901 and MGC-803 cells with si-FEZF1-AS1 or pcDNA-FEZF1-AS1. GAPDH protein was used as an internal control

Dysregulation of cell cycle is a vital reason for tumor cell proliferation, to further explore whether *FEZF1-AS1* promoted proliferation by regulation cell cycle progression *in* gastric cancer cell; we examined cell cycle by using flow cytometric analysis. The results revealed that SGC-7901 and AGS cells with si-RNAs had an obvious cell cycle arrest in the G1–S phase and the population of cells in the S phase was decreased (Fig. 4a and b). However, ectopic expression *FEZF1-AS1* induced G1-S progression and accumulated S phase (Fig. 4c). Furthermore, we examined apoptosis rate by using flow cytometric analysis. The results showed that the percentage of early and later apoptotic cells was significantly increased in SGC-7901 and AGS cells with si- *FEZF1-AS1* than the si-NC cells (Fig 4d and e). Whereas ectopic expression *FEZF1-AS*1 repressed apoptosis than pcDNA vector (Fig. 4f). Moreover, western blot analysis showed that the protein levels of CyclinD1/CDK2/CDK4/CDK6 were significantly decreased in AGS and SGC-7901cell with si- RNA (Fig 4g and h); the result was conserved in MGC-803cell (Fig. 4i). These results confirmed that *FEZF1-AS1* is involved in cell-cycle regulation.

### FEZF1-AS1 downregulated P21 expression driving cell cycle

**Fig. 5:**
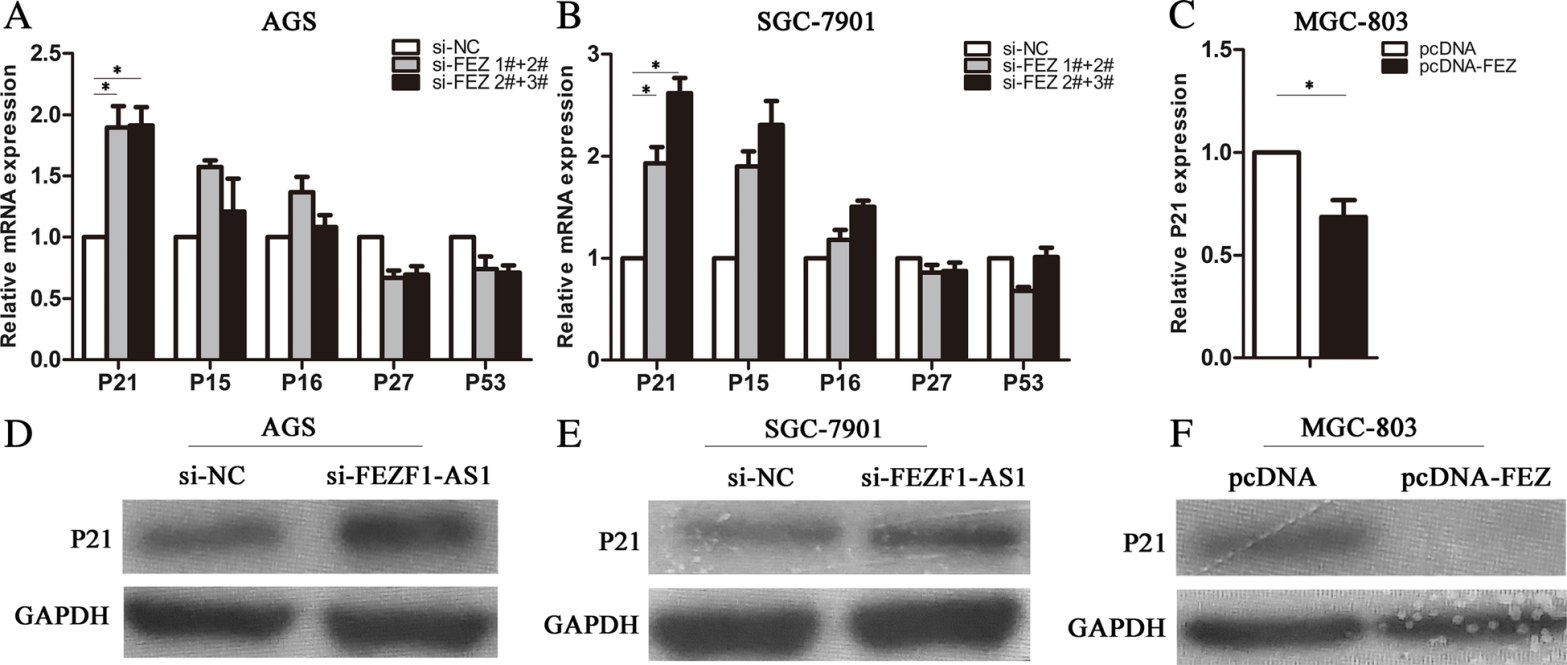
*FEZF1-AS1 downregulated P21 expression driving cell cycle.* **a**, **b** and **c** QRT–PCR was used to detect mRNAs expression of AGS, SGC-7901 and MGC-803 cells with *si-FEZF1-AS1* or pcDNA-FEZF1-AS1. Values represented the mean ± SD, from three independent experiments *P < 0.05, **P < 0.01. **d**, **e** and **f** Western blot analysis of P21 in AGS, SGC-7901and MGC-803 cells with si-FEZF1-AS1 or pcDNA-FEZF1-AS1. GAPDH protein was used as an internal control

To investigate whether *FEZF1-AS1* could regulate cell-cycle, Additional file 3: Figure S2A illustrates plots from gene set enrichment analysis (GSEA) using gastric cancer patient gene profiling data (GSE53137) showing that gene set differences in *FEZF1-AS1* high vs. low patients indicated that *FEZF1-AS1* regulates gene sets mainly associated with cell cycle progression. Cyclin dependent kinase inhibitor (CKI) was an important cell cycle regulator and P21 was one of the most important downstream target genes of tumor suppressor P53. Next, to investigate whether these genes could be regulated by *FEZF1-AS1* in gastric cancer cells, we subsequently detected mRNAs and proteins in AGS and SGC7901 cells with si- *FEZF1-AS1* and MGC-803 cell with pcDNA-FEZF1-AS1. The results showed that p21expression was up-regulated by2.54 fold and 2.07 fold compared with control cells (P < 0.01, Fig. 5a and b). However, ectopic expression of *FEZF1-AS1* downregulated P21 expression than pcDNA vector (Fig. 5c). Furthermore, Weston blot assays showed that protein levels of p21was significantly increased in SGC-7901and AGS cell with si-*FEZF1-AS1* cells (Fig. 5d and e); however, erogenous *FEZF1-AS1* expression decreased the protein levels of p21 than pcDNA vector (Fig. 5f). Next, we investigated the role of *P21* in *FEZF1-AS1* promoted proliferation. Weston blot assays showed that protein levels of CDK2/CDK4/CDK6 was significantly decreased in SGC-7901 with si-FEZF1-AS1 cells; however, co-transfect of si-FEZF1-AS1 and si-P21 partly reversed CDK2/CDK4/CDK6 expression than si-NC (Additional file: 4 Figure S3A). These data indicated that P21 was involved in *FEZF1-AS1-*regulated cell cycle, contributing to gastric cancer cells proliferation.

### FEZF1-AS1epigenetically silenced P21 transcription through LSD1-Mediated H3K4me2 demethylation

**Fig. 6:**
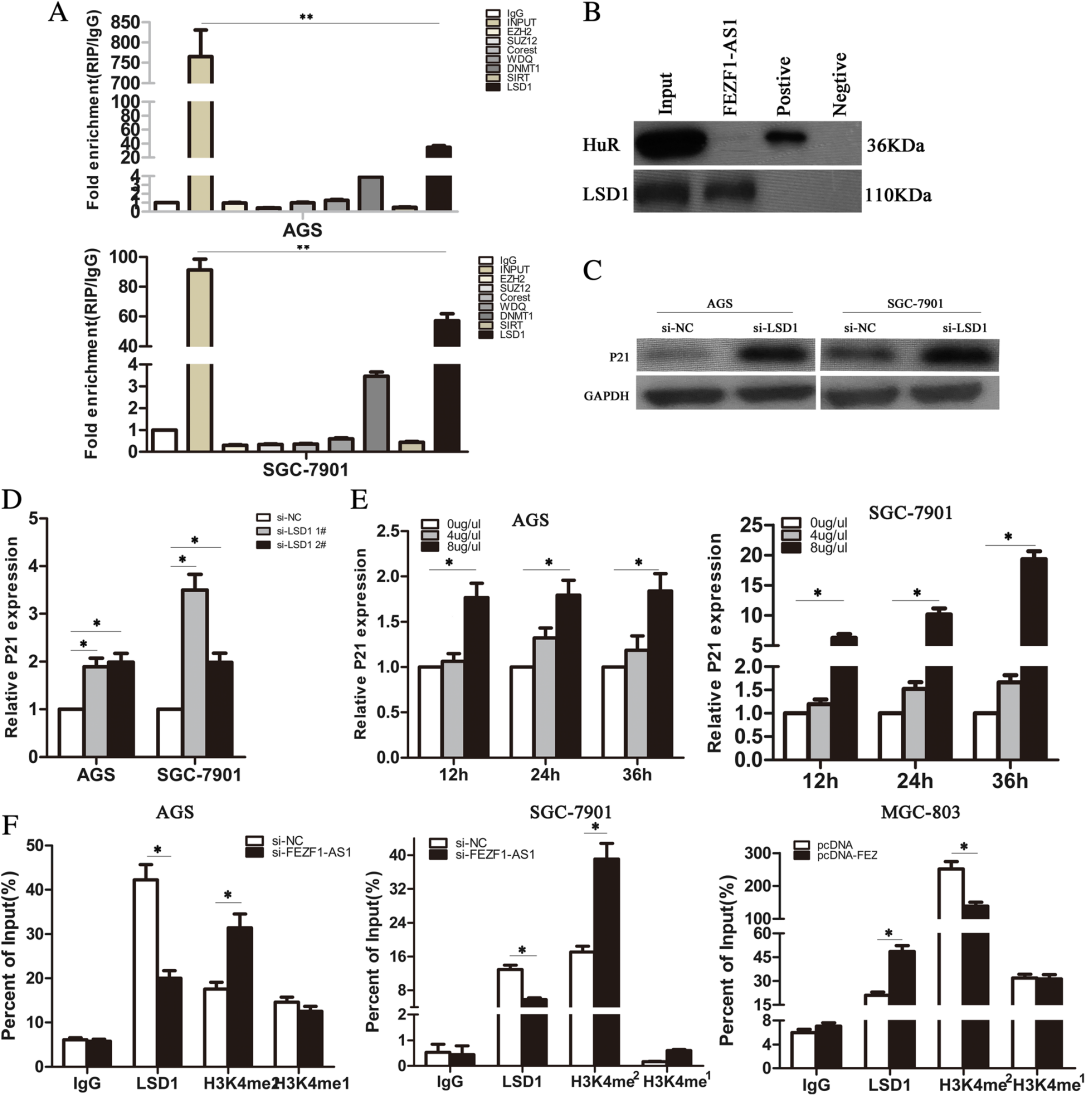
*FEZF1-AS1epigenetically silenced P21 transcription through LSD1-Mediated H3K4me2 demethylation.* **a** RIP experiments were performed using the LSD1, EZH2, SUZ12, WDR5, coREST antibodies for immunoprecipitation. Specific primers for *FEZF1-AS1* were used to detect *FEZF1-AS1*. **b** Biotinylated *FEZF1-AS1* or antisense RNA was incubated with total-cell extracts (SGC7901 cells), targeted with streptavidin beads and washed; the associated proteins were resolved in a gel. Western blotting analysis of the specific association of LSD1 as well as UPF1 with *FEZF1-AS1*. A nonspecific protein (GAPDH) is shown as a control. **c**, **d** Expression of P21 in AGS, SGC-7901 and MGC-803 cells with si-LSD1 or pcDNA-FEZF1-AS1was detected by qRT–PCR and Western blotting. **e** Expression of P21 in AGS and SGC-7901 cells with inhibtor of LSD1 was detected by qRT–PCR. **f** ChIP analyses in AGS, SGC-7901and MGC-803cells with si-FEZF1-AS1 or pcDNA-FEZF1-AS1 were performed on the P21 promoter regions using anti-H3K4me1, H3K4me2 and LSD1 antibodies. Enrichment was determined relative to the input controls. All experiments were performed in triplicate with three technical replicates.*P < 0.05 and **P < 0.01

To further explore the molecular mechanisms by which *FEZF1-AS1* regulated P21 transcription, we used ENCODE Histone Modification Tracks embedded in UCSC Genome Browser and found H3K4me2 enrichment peaks in the P21 promoter region (Additional file 3: Figure S2B and 2D). Considering that mechanisms of lncRNAs largely depend on specific cell locations, we found *FEZF1-AS1* RNA was mostly located in the nucleus versus the cytoplasm (Additional file 3: Fig S2C), thus suggesting *FEZF1-AS1* may exert transcriptional regulation function. Next, we conducted RIP assays and RNA-pull down assays to examine FEZF1*-AS1*’s binding protein. As shown in Fig. 6a, the endogenous *FEZF1-AS1* was enriched in the anti-LSD1 RIP fraction in AGS and SGC-7901 cells. Differential protein LSD1was specifically precipitated by *FEZF1-AS1* in RNA-pull-down assay (Fig. 6b). Lysine-specific demethylase 1 (LSD1), the first Lysine demethylase identified, which demethylates mono- and di-methylated residues of lysine-4 on histone H3 (H3K4me1, H3K4me2 orH3K9me1), and LSD1could promote neural stem cell proliferation [36]. To further explore the molecular mechanisms of *FEZF1-AS1* regulating P21 through LSD1-Mediated demethylation. Next, we knocked down LSD1 by si-RNA in AGS and SGC-7901 cells, and demonstrated that mRNA and protein of P21 was upregulated compared to the controls (Fig. 6c and d); moreover, P21was enhanced in AGS and SGC-7901 cells treated with the LSD1inhibitor compared to untreated (Fig. 6e). Furthermore, co-transfect of pcDNA-FEZF1-AS1 and si-LSD1 partly reversed P21 expression than pcDNA vector (Additional file 4: Figure S3B). These results demonstrated that *FEZF1-AS1* may directly bound with SD1 and possiblely regulated expression of P21 in the transcriptional level. Next, we used ChIP assays to verificate the mechanism. We analyzed the ChIP assays LSD1, dymethylation of histone H3 on lysine-4(H3K4me1 and H3K4me2), markers which are associated with transcriptional regression on P21 promoter in presence of si-FEZF1-AS1. The results shown that LSD1 could directly bind to the promoter region of *P21* and mediate H3K4me2 modification, while knockdown of *FEZF1-AS1* led to reduced LSD1 and increased H3K4me2 demethylation ability (Fig. 6 f); however, H3K4me1 was no change.

In conclusion, these data indicated that *FEZF1-AS1* recruit the LSD1 to repress P21 transcription via H3K4me2 modification.

### Transcription factor SP1was involved in the upregulation of FEZF1-AS1

**Fig. 7:**
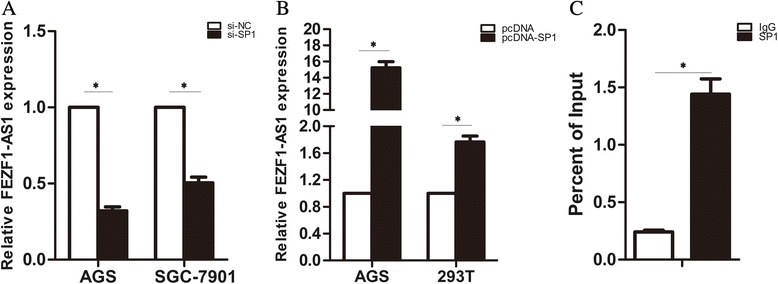
*Transcription factor SP1was involved in the upregulation of FEZF1-AS1.* **a** Expression of *FEZF1-AS1* in AGS and SGC-7901with si-SP1 was detected by qRT–PCR. **b** Expression of *FEZF1-AS1* in AGS and 293 T with pcDNA-SP1 was detected by qRT–PCR. **c** ChIP assays were used to show direct binding of SP1 to endogenous *FEZF1-AS1* promoter regions (below). Bars: SD.; **P < 0.01

Relative expression levels of *FEZF1-AS1* were overexpressed in gastric cancer cells compared to GES-1 cells. Then we explored the reason of overexpression of FEZF1-AS1. Abnormal of expression lncRNA are regulated by transcription factors and epigenetic modification, then we used the JASPAR software to analysis the promoter of FEZF1-AS1,which includes transcription factors SP-1 (Table 2). Next, we detected the expression of *FEZF1-AS1* in gastric cancer cells with si-SP1, pcDNA-SP1 and control, the results shown that relative expression of *FEZF1-AS1*was downregulated in AGS and SGC-7901cells with si-SP1 (Fig. 7a); however, expression of *FEZF1-AS1*was upregulated in AGS and 293 T cells with pcDNA-SP1 compared with pcDNA vector (Fig. 7b). We used ChIP assays to determine that SP1 band to the endogenous *FEZF1-AS1* promoter. The results of ChIP assays showed that SP1 could directly bind to *FEZF1-AS1* promoter regions and induce *FEZF1-AS1* transcription in AGS and SGC-7901 cells (Fig. 7c). Above results demonstrated overexpression of *FEZF1-AS1* is mechanistically linked to increased gastric cancer cell proliferation via dependence on SP1. Finally, correlation analysis revealed that *FEZF1-AS1*expression levels were positive correlation with SP1 and CDK2/CDK4/CDK6/CyclinD1 and inversely correlated with P21 expression levels in Gastric Cancer tissues (Additional file 5: Figure S4). These results indicate that *FEZF1-AS1* overexpression upregulated CDK2/CDK4/CDK6/CyclinD1expression by suppression P21 expression*.*

## Discussion

Over the past decades, mounting evidences have emphasized the emerging significance of lncRNAs in diverse human cancer, including gastric cancer [19, 37, 38]. Forthrtmore, a small part of the study has shown that lncRNA expression profiles is predicting cancer or discriminating between cancer subtypes. In fact, lncRNAs have an obvious merit of their relative tissue-specific expression and functional layout as transcriptional levels. lncRNAs may better reflect the biologic status of cancer cells. However, lncRNAs in gastric cancer are still an emerging field, only a few of lncRNAs have been characterized in gastric cancer tumorigenesis and should be further studied as predictive biomarkers. One of these lncRNAs is gastric adenocarcinoma predictive long intergenic noncoding RNA (*GAPLINC*), GAPLINC is overexpression and a predictive marker for metastasis and prognosis in gastric cancer [39]. In this report, we found that the expression of another lncRNA, FEZF1-AS1, was significantly upregulated in gastric cancer tissues, and was correlated with poor prognosis. Furthermore, we have presented a study for the prediction of cancer/normal tissues and biomarkers using FEZF1-AS1 expression, suggesting that FEZF1-AS1 may be an independent clinical marker in gastric cancer diagnosis and prognosis.

The dysregulation of lncRNAs joins a wide variety of pathological processes, but the mechanisms of lncRNAs expression are not clear and further exploration is required. Transcription factor and epigenetic regulatory factors could manipulate the expression of lncRNAs [40, 41]. Here, through bioinformation analysis, we found that FEZF1-AS1promoter contained conserved SP1-binding site, which is a vital transcription factor in sustaining the “hall markers” of cancer [42]. Accumulating data has revealed that SP1 is overexpressed in breast cancer and gastric cancer [43, 44]. Our findings evidenced that SP1 is a key factor in controlling FEZF1-AS1 expression. These results, along with those recent studies [45, 46], underline the role of transcription factors in regulating lncRNA transcription.

Additionally, our data demonstrated that knockdown FEZF1-AS1expression contributed to significant inhibition of cell proliferation both in vitro and in vivo, whereas exogenous expression FEZF1-AS1 led to cell growth. Downregulation FEZF1-AS1 expression caused G1 phase arrest and S phase reduction suppressing cell cycle progression. The G1–S transition in the cell cycle in mammalian cells is controlled by cyclins, cyclin-dependent kinases (CDKs) and their inhibitors, and the deregulation of CKIs is a common feature in tumor cells [47]. P21, one of the most CKIs, is important checkpoints of P53 signaling pathway for G1/S transition by inhibiting the activity of kinases such as CyclinD/CDK4, CyclinD/CDK6 and CyclinE/CDK2 [48, 49], which plays multiple roles in inhibition cell proliferation in normal and cancer cells and was almost downregulated in many types of cancer. Notably, we found that P21 was remarkably upregulated upon FEZF1-AS1 knockdown. Our findings demonstrated that FEZF1-AS1 mediated gastric cancer cell proliferation promotion, which possibly also downregulated p21 expression.

A small number of functional lncRNAs have been well characterized, which can regulate gene expression at various levels, including chromatin modification, transcription and post-transcriptional processing. lncRNAs can act as molecular decoys binding and titrating away proteins or RNAs to indirectly exert biological functions in multiple kingdoms of life. HOTAIR is one of the most studied lncRNAs involved in chromatin modification, which can recruit PRC2 genome-wide to alter H3K27 methylation and gene expression patterns. lncRNA MALAT1 could bind to SFPQ to release PTBP2 from the SFPQ/PTBP2 complex and increase SFPQ-detached PTBP2 promoting CRC cell proliferation and migration [50]. In addition, lncRNAs can recruit chromatin-modifying enzymes to target genes by acting as guides, either in cis (near the site of lncRNA production) [51] or in trans to distant target genes [52]. In this study, the results of RNA and RNA-pull-down assays show that FEZF1-AS1 could bind with LSD1, the first discovered histone demethylase. LSD1 participate in development and differentiation regulation of chromatin remodeling and histone demethylation, which could specifically catalysed the demethylation of mono- and di-methylated histone H3 lysine 4(H3K4) and H3 lysine 9 (H3K9) through a redox process. More importantly, overexpression of LSD1 is involved in many pathological processes of cancer, such as proliferation, apoptosis and metastasis of various cancer cells [26, 28, 34]. S. Lim et al. reported [34] that knockdown LSD1 significantly reduced levels of H3K9me2 at the p21 locus regression cell proliferation through regulation of cell cycle. Our study demonstrated that knockdown FEZF1-AS1 led to enhance levels of H3K4me2 at the p21 promoter and a nearly unchanged H3K4me1 levels.

## Conclusions

**Fig. 8:**
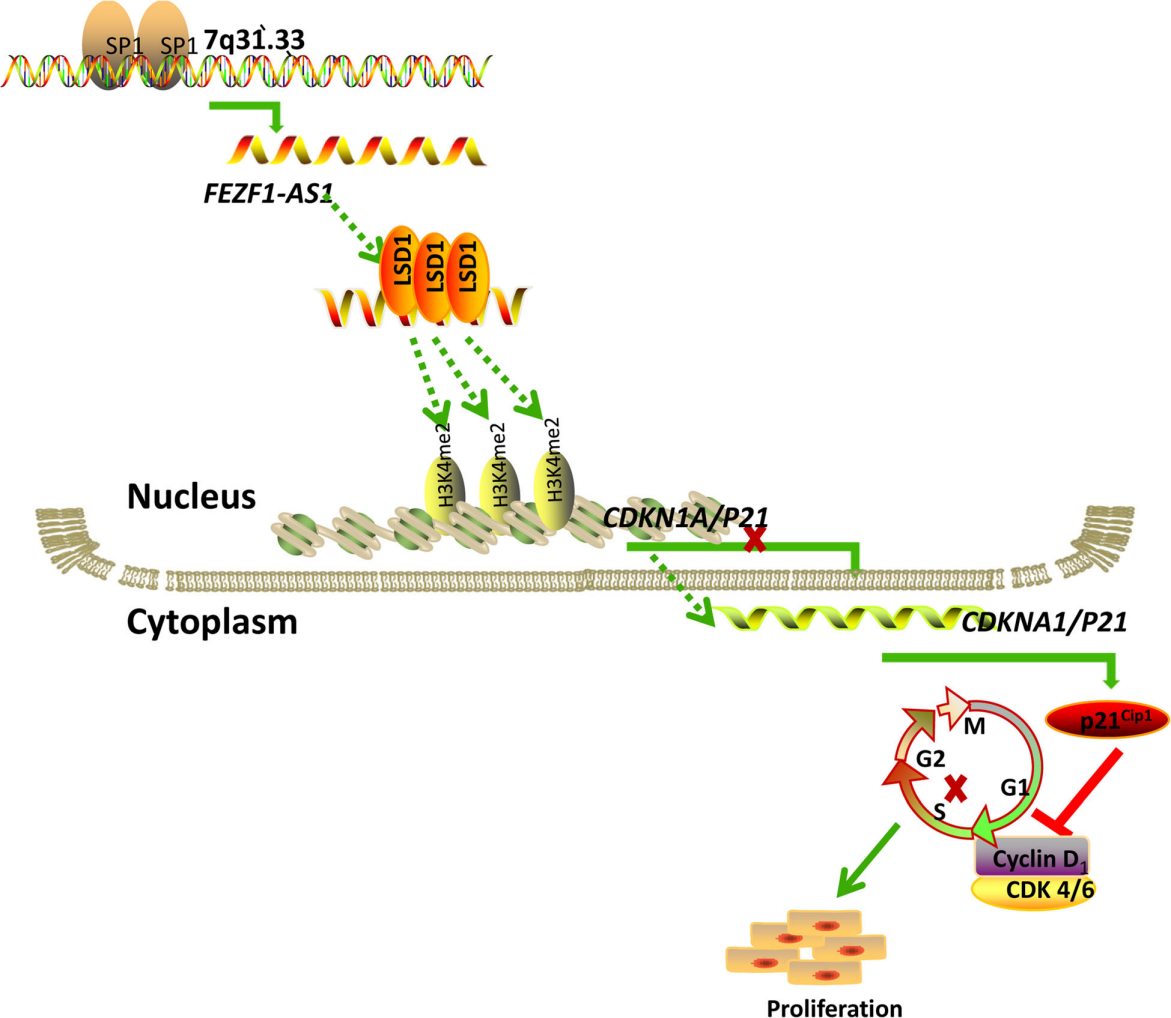
Summary diagram described that *FEZF1-AS1*regulates GC cell proliferation: (I)SP1 accelerated *FEZF1-AS1*overexpressioon in gastric cancer; (II) *FEZF1 -AS1* inhibited transcription of P21 to cause G1-S arrest contributing to proliferation; (III) *FEZF1 -AS1* recruited and binded to LSD1 demethylation H3K4me2 at the p21 promoter regulation P21expression in gastric cancer

In this study we had evidenced that FEZF1-AS1 was overexpressed in gastric cancer tissues; its overexpression may predict poor prognosis. FEZF1-AS1 promoted gastric cancer cell proliferation and tumorigenesis in vivio and vivo by affecting cell cycle progression. In addition, we described the molecular mechanism by which FEZF1-AS1 boost gastric cancer cell proliferation (Fig. 8) :(I) SP1 accelerated FEZF1-AS1overexpressioon in gastric cancer; (II) FEZF1 -AS1 caused G1-S arrest contributing to proliferation; (III) FEZF1 -AS1 repressed p21 transcription by recruiting LSD1 causing H3K4me2 demethylation at the p21 promoter in gastric cancer. Finally, these data provided new insights into the RNA regulation network, indicating that lncRNAs could target chromatin-modifying enzymes regulating gene expression; LncRNAs have been proposed as potential targets for prognosis and therapeutic intervention.

## Additional files

Additional file 1: Table S1. Primer sequences. (XLSX 8 kb)
Additional file 2: Figure S1. (**A**) The endogenous expression of *FEZF1-AS1* of gastric cancer cell lines (SGC-7901, MGC-803, BGC-823, AGS, HGC-27) and GES-1 cell. (**B**) QRT–PCR was used to detect *FEZF1-AS1* expression of AGS, SGC-7901 and MGC-803cells with si-FEZF1-AS1 or pcDNA-FEZF1-AS1. (**C**) QRT–PCR was used to detect *FEZF1-AS1* expression of SGC-7901 cells with sh- FEZF1-AS1. (**D**) QRT–PCR was used to detect SP1 expression of AGS, SGC-7901 and 293 T cells with si-SP1 or pcDNA-SP1. All experiments were performed in triplicate with three technical replicates. (TIF 915 kb)
Additional file 3: Figure S2. (**A**) The GSEA results showed enrichment of several genes that may be regulated by *FEZF1-AS1. FEZF1-AS1* had significantly negative correlation with genes involved in cell cycle arrest in gastric cancer dataset(GSE15459). The barcode plot indicates the position of the genes in each gene set, and red and blue represent positive or negative Pearson’s correlation with *FEZF1-AS1* expression, respectively. (**B**) The RNA binding proteins with *FEZF1-AS1* by GEO DataSet analysis. (**C**) Genome Browser and analyzed H3K4 enrichment peaks in the *P21* promoter region. (**D**) *FEZF1-AS1* expression levels in cell nucleus or cytoplasm of AGS, SGC-7901 and MGC-803 cells were detected by qRT-PCR. U6 was used as a nucleus marker and GAPDH was used as a cytosol marker. (TIF 7608 kb)
Additional file 4: Figure S3. (**A**) Proteins expression of CDK2/CDK4/CDK6 in SGC-7901 cells with si-FEZF1-AS1 or si-P21 were detected by Western blotting. (**B**) Proteins expression of P21 in MGC-803 cells with si-LSD1 or pcDNA-FEZF1-AS1 were detected by Western blotting. (TIF 757 kb)
Additional file 5: Figure S4. The expressions of P21/SP-1/CDK2/CDK4/CDK6/CyclinD1 and correlation with expressions of FEZF1-AS1 in 40 gastric cancer tissues (**A**) The expression of SP-1 was positive correlation with expressions of FEZF1-AS1 in 40 gastric cancer tissues(R^2^ = 0.197, *P =* 0.0042). (**B**) The expression of P21 was negative correlation with expressions of FEZF1-AS1 in 40 gastric cancer tissues (R^2^ = 0.154, *P =* 0.0124). (**A**) The expressions of CDK2/CDK4/CDK6/CyclinD1 were positive correlation with expressions of FEZF1-AS1 in 40 gastric cancer tissues, respectively (R^2^ = 0.111, *P =* 0.0358; R^2^ = 0.184, *P =* 0.0058; R^2^ = 0.144, *P =* 0.0159 ; R^2^ = 0.122, *P =* 0.269). (TIF 504 kb)

## Abbreviations

BSA: Bovine serum albumin
CDK: Cyclin-dependent kinase
CDK2: Cyclin-dependent kinase 2
CDK4: Cyclin-dependent kinase 4
CDK6: Cyclin-dependent kinase 6
ChIP: Chromatin immunoprecipitation
CKI: Cyclin dependent kinase inhibitor
CT: Cycle threshold
DFS: Disease-free survival
DMEM: Dulbecco’s modified eagle medium
ECM: Extracellular matrix
EMT: Epithelial-to- mesenchymal transition
EZH2: Enhancer of zest homolog 2
GAPDH: glyceraldehyde-3-phosphate dehydrogenase
GEO: Gene expression omnibus
GSEA: Gene set enrichment analysis
H3K27me3: Histone H3lysine-27 trimethylation
H3K4me1: Histone H3lysine-4 mono-methylation
H3K4me2: Histone H3lysine-4 di -methylation
H3K9me2: Histone H3lysine-9 di -methylation
HE: Hematoxylin and eosin
HOTAIR: HOX antisense intergenic RNA
IHC: Immunohistochemistry
LncRNA: Long non-coding RNA
LSD1: Lysine-specific demethylase 1
miR34a: microRNA34a
ncRNA: Non-coding RNA
PBS: Phosphate-buffered saline
PcG: Polycomb group protein
PMSF: Phenylmethanesulfonyl fluoride
PRC2: Polycomb repressive complex 2
PVDF: Polyvinylidene fluoride
qRT-PCR: Quantitative real-time polymerase chain reaction
RIP: RNA immunoprecipitation
ROC: Receiver operating characteristic
SDS-PAGE: Sodium dodecyl sulfate-polyacrylamide gel electrophoresis
ShRNA: Short hairpin RNA
SiRNA: Small interfering RNA
TBS: Tris-buffered saline
UICC: Union for international cancer control
WHO: the World Health Organization
