# Supplementary figures and images for: LincRNAFEZF1-AS1 represses p21 expression to promote gastric cancer proliferation through LSD1-Mediated H3K4me2 demethylation
Source: Mol Cancer. 2017 Feb 16;16:39. doi: 10.1186/s12943-017-0588-9 (PMC5314465; doi:10.1186/s12943-017-0588-9)

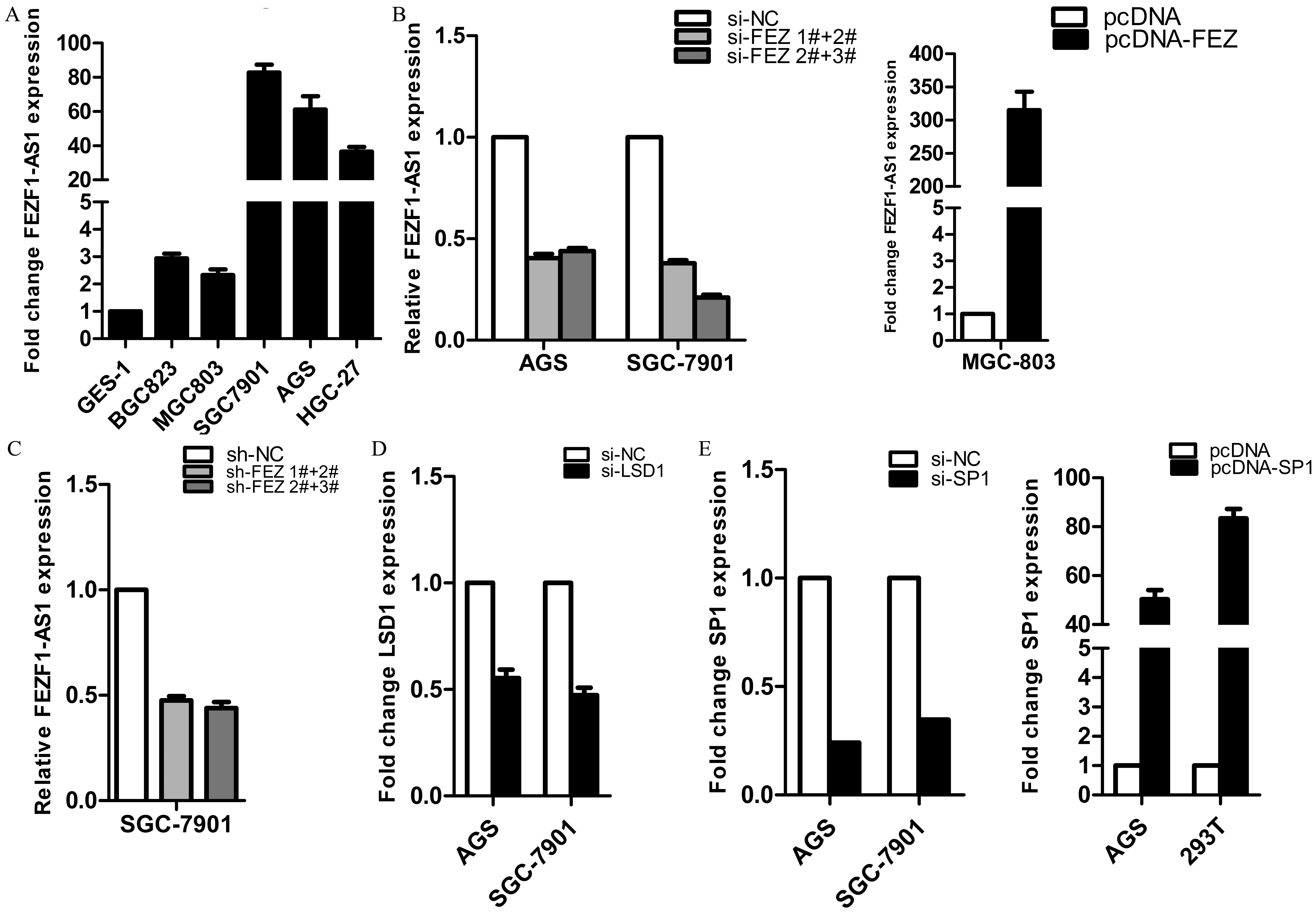

Supplement: Additional file 2: Figure S1. — (A) The endogenous expression of FEZF1-AS1 of gastric cancer cell lines (SGC-7901, MGC-803, BGC-823, AGS, HGC-27) and GES-1 cell. (B) QRT–PCR was used to detect FEZF1-AS1 expression of AGS, SGC-7901 and MGC-803cells with si-FEZF1-AS1 or pcDNA-FEZF1-AS1. (C) QRT–PCR was used to detect FEZF1-AS1 expression of SGC-7901 cells with sh- FEZF1-AS1. (D) QRT–PCR was used to detect SP1 expression of AGS, SGC-7901 and 293 T cells with si-SP1 or pcDNA-SP1. All experiments were performed in triplicate with three technical replicates. (TIF 915 kb) [file 12943_2017_588_MOESM2_ESM.tif]

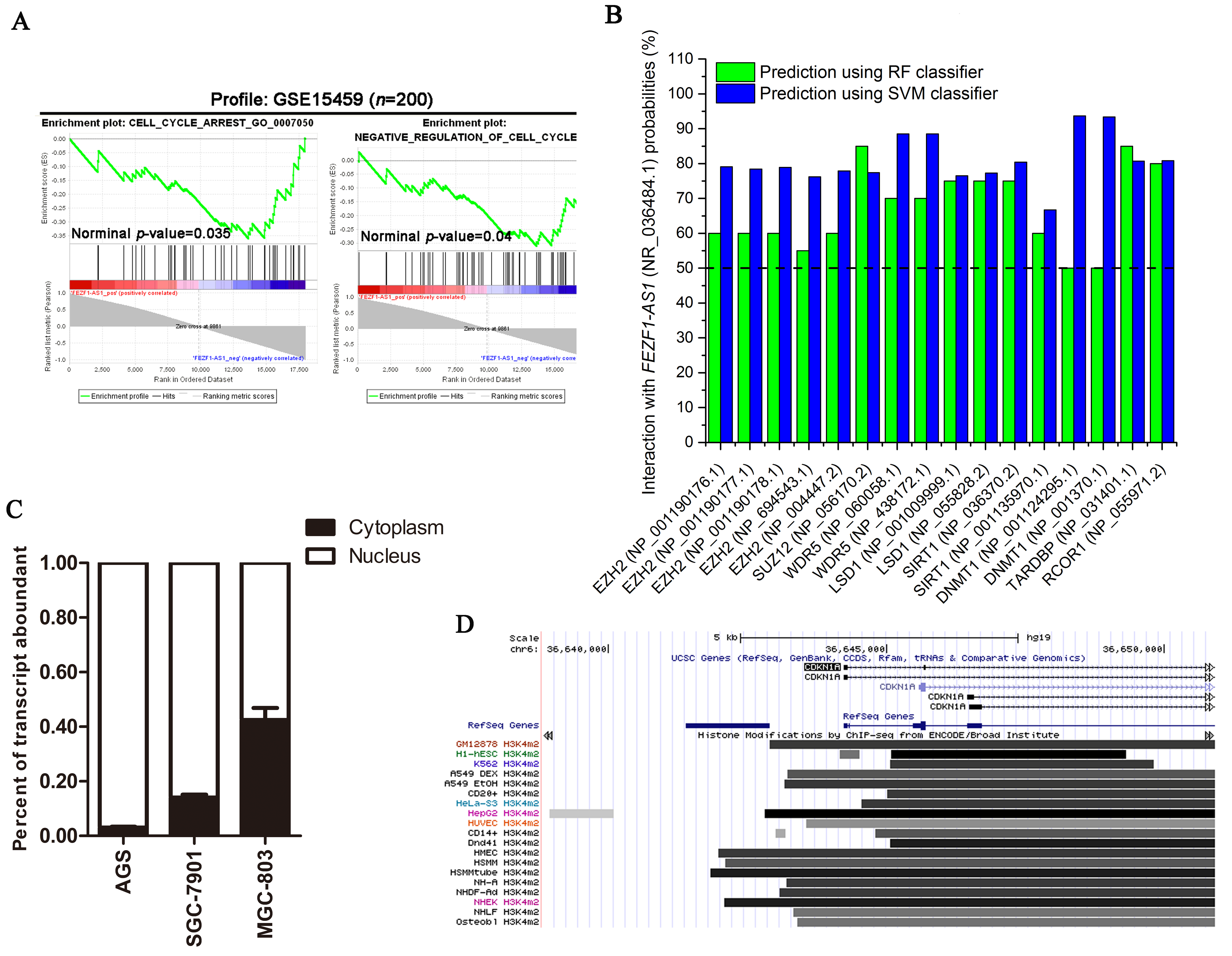

Supplement: Additional file 3: Figure S2. — (A) The GSEA results showed enrichment of several genes that may be regulated by FEZF1-AS1. FEZF1-AS1 had significantly negative correlation with genes involved in cell cycle arrest in gastric cancer dataset(GSE15459). The barcode plot indicates the position of the genes in each gene set, and red and blue represent positive or negative Pearson’s correlation with FEZF1-AS1 expression, respectively. (B) The RNA binding proteins with FEZF1-AS1 by GEO DataSet analysis. (C) Genome Browser and analyzed H3K4 enrichment peaks in the P21 promoter region. (D) FEZF1-AS1 expression levels in cell nucleus or cytoplasm of AGS, SGC-7901 and MGC-803 cells were detected by qRT-PCR. U6 was used as a nucleus marker and GAPDH was used as a cytosol marker. (TIF 7608 kb) [file 12943_2017_588_MOESM3_ESM.tif]

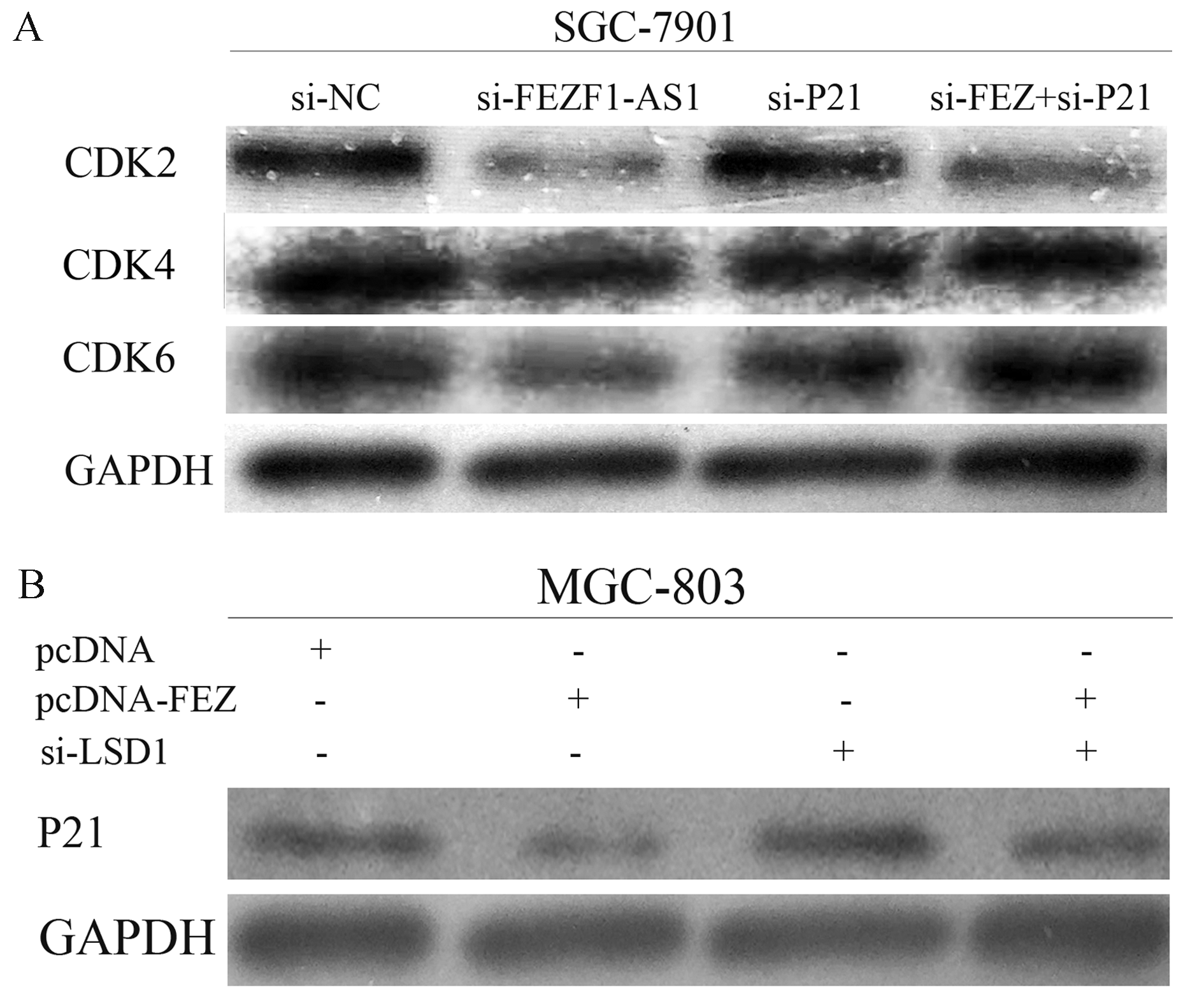

Supplement: Additional file 4: Figure S3. — (A) Proteins expression of CDK2/CDK4/CDK6 in SGC-7901 cells with si-FEZF1-AS1 or si-P21 were detected by Western blotting. (B) Proteins expression of P21 in MGC-803 cells with si-LSD1 or pcDNA-FEZF1-AS1 were detected by Western blotting. (TIF 757 kb) [file 12943_2017_588_MOESM4_ESM.tif]

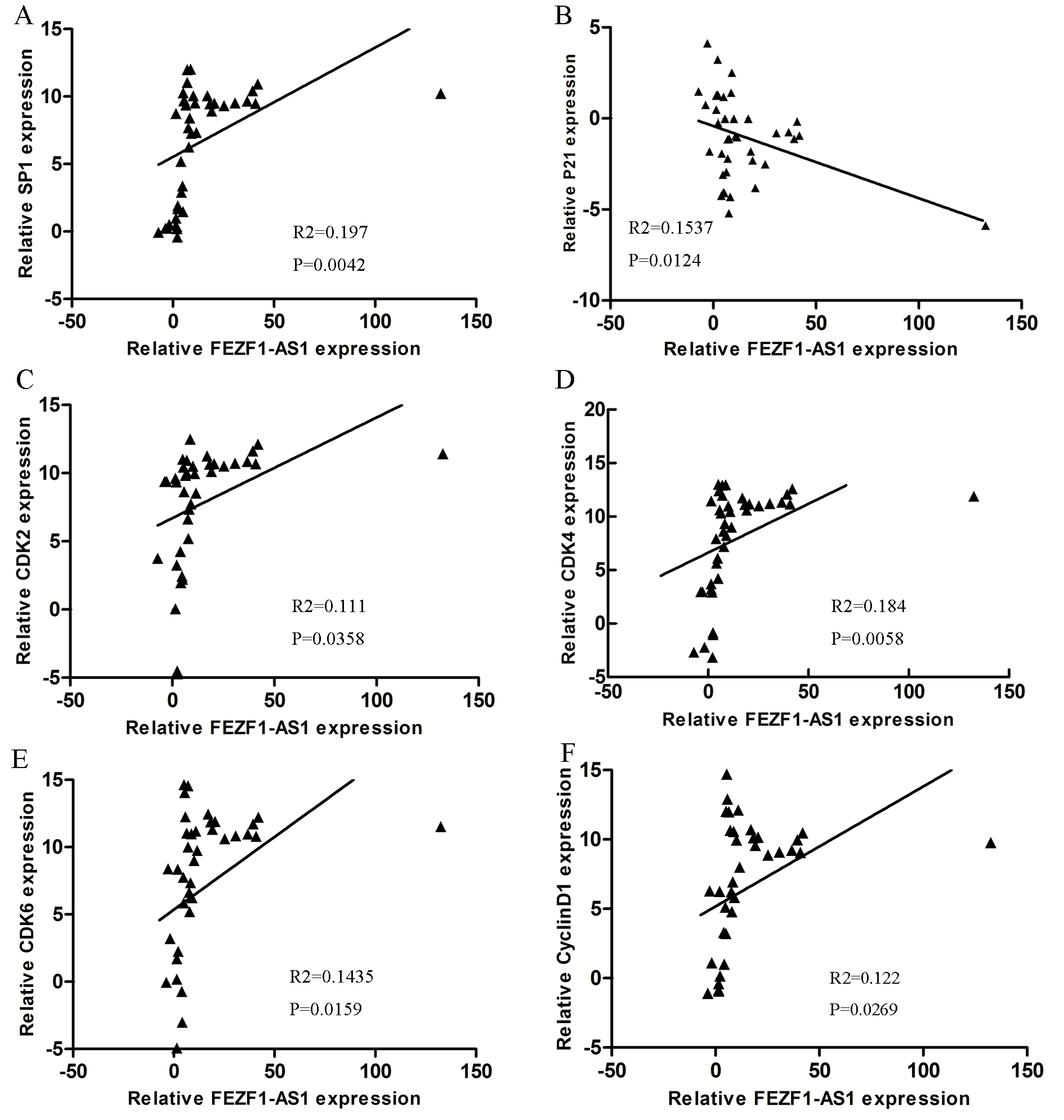

Supplement: Additional file 5: Figure S4. — The expressions of P21/SP-1/CDK2/CDK4/CDK6/CyclinD1 and correlation with expressions of FEZF1-AS1 in 40 gastric cancer tissues (A) The expression of SP-1 was positive correlation with expressions of FEZF1-AS1 in 40 gastric cancer tissues(R2 = 0.197, P = 0.0042). (B) The expression of P21 was negative correlation with expressions of FEZF1-AS1 in 40 gastric cancer tissues (R2 = 0.154, P = 0.0124). (A) The expressions of CDK2/CDK4/CDK6/CyclinD1 were positive correlation with expressions of FEZF1-AS1 in 40 gastric cancer tissues, respectively (R2 = 0.111, P = 0.0358; R2 = 0.184, P = 0.0058; R2 = 0.144, P = 0.0159 ; R2 = 0.122, P = 0.269). (TIF 504 kb) [file 12943_2017_588_MOESM5_ESM.tif]
